# Predicting the seed microbiome using phylogeny-driven machine learning

**DOI:** 10.1186/s40793-026-00936-1

**Published:** 2026-07-13

**Authors:** Julia Herbinger, Dinesh Kumar Ramakrishnan, Jannik Reißfelder, Majharulislam Babor, Marina Höhne, Ahmed Abdelfattah

**Affiliations:** 1 Department of Data Science, Leibniz Institute for Agricultural Engineering and Bioeconomy (ATB), Max-Eyth-Allee 100, 14469 Potsdam, Germany; 2 Department of Microbiome Biotechnology, Leibniz Institute for Agricultural Engineering and Bioeconomy (ATB), Max-Eyth-Allee 100, 14469 Potsdam, Germany; 3 Institute for Biochemistry and Biology, University of Potsdam, Karl-Liebknecht-Str. 24/25, 14476 Potsdam, Germany; 4 Department of Computer Science, University of Potsdam, An Der Bahn 2, 14476 Potsdam, Germany

**Keywords:** Co-evolution, Phylosymbiosis, Seed microbiome, Microbial inheritance, Vertical transmission, Machine learning, Microbiome prediction

## Abstract

**Background:**

The composition of the seed-associated bacterial microbiome can reflect host evolutionary relationships, a pattern consistent with phylosymbiosis. While machine learning offers new opportunities to predict microbial community composition, existing models often require prior microbial profiles or environmental variables, limiting their application to unsampled hosts. Here, we tested whether plant nuclear internal transcribed spacer (ITS) sequences, used as a marker of host relatedness, can predict species-level seed-associated bacterial communities using 16S rRNA data from 61 plant species.

**Results:**

We introduced customized machine learning models that use sequence-based Hamming distances to capture plant host relatedness. Among the tested models, the Hamming Distance-based k-Nearest Neighbor model (HD-KNN) achieved the highest overall predictive accuracy, yielding an average Jensen-Shannon divergence (JSD) of 0.276 between observed and predicted microbiome profiles. HD-KNN performed particularly well within densely sampled host groups, including *Brassicaceae* and *Poaceae*, where closely related reference species were available. In contrast, Hamming Distance-based Gaussian Process Regression (HD-GPR) showed slightly better performance for phylogenetically isolated species, suggesting that model performance depends on host representation within the training dataset.

**Conclusions:**

Our framework demonstrates that plant nuclear ITS-derived host relatedness carries a partial predictive signal for seed-associated bacterial microbiome composition. These results provide a foundation for low-input predictive modelling of seed-associated bacteria and may help prioritise microbiome predictions for unsampled plant species when closely related reference species are available. However, our conclusions are strictly limited to seed-associated bacterial communities and should not be directly generalized to fungal communities or other plant compartments, such as the rhizosphere or phyllosphere, which may be shaped by different environmental filtering mechanisms.

**Supplementary Information:**

The online version contains supplementary material available at 10.1186/s40793-026-00936-1.

## Introduction

**Fig. 1:**
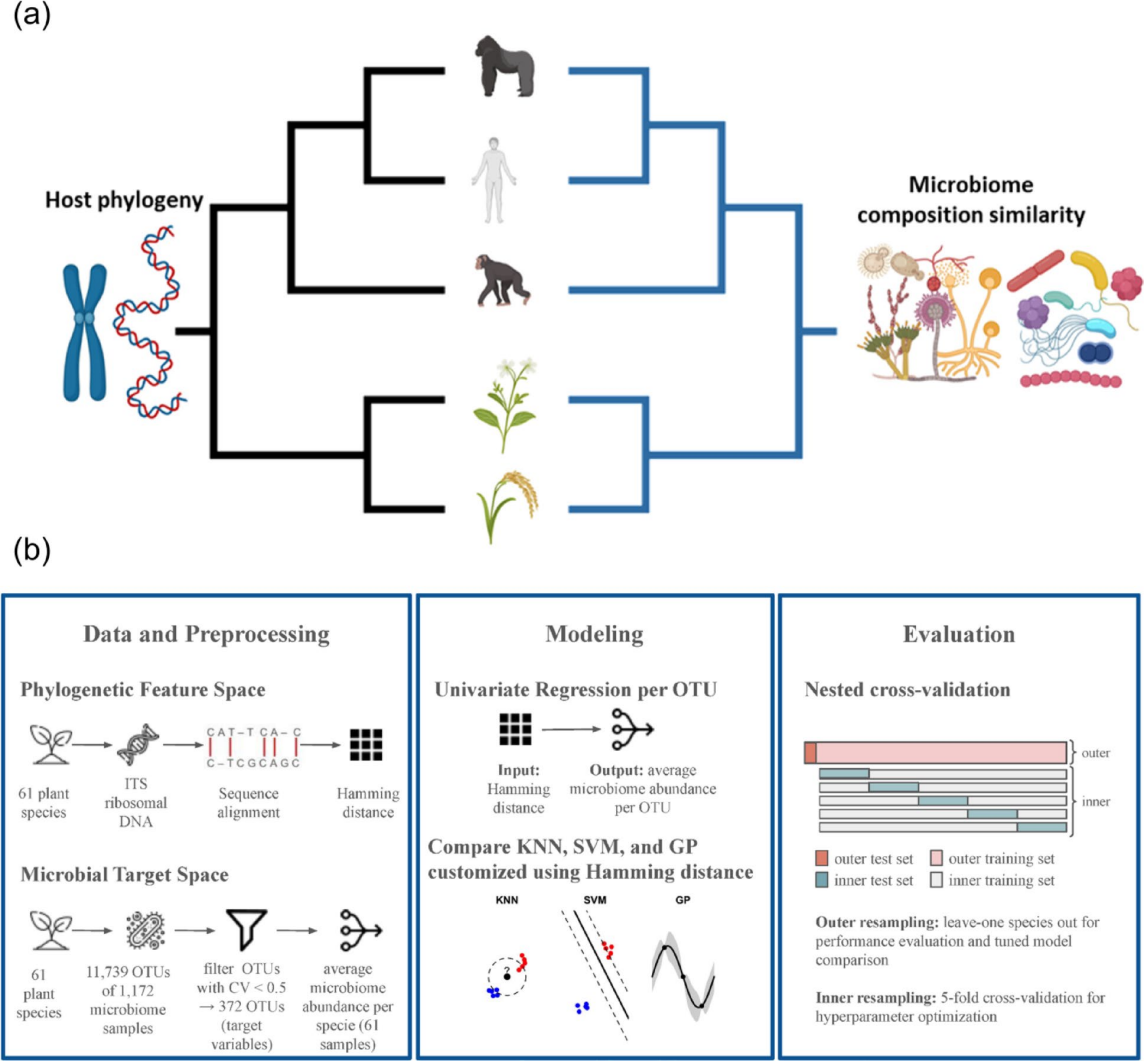
Conceptual and methodological framework for phylogeny-based plant microbiome prediction. **a** Conceptual representation of phylosymbiosis, where microbiome composition reflects host phylogeny. Microbial communities tend to be more similar among closely related plant species than between distantly related hosts, such as those from different kingdoms. **b** Overview of the workflow: plant phylogenetic features were derived from plant nuclear ribosomal ITS sequence alignments using normalized Hamming distances; microbial target variables were filtered OTUs from seed microbiomes; and customized machine learning models (HD-KNN, HD-GPR, HD-SVR) were trained via univariate regression per OTU using nested cross-validation to evaluate predictive performance. Figure 1a created in BioRender. Ramakrishnan, D. (2025) https://BioRender.com/jiy3u5n

The plant microbiome has a significant role in plant health, growth, tolerance to biotic and abiotic stresses [1–3]. While the composition of the microbial community associated with plants is influenced by several factors [4, 5], species identity is one of the main determinant of the plant microbiome [6, 7]. In fact, the divergence in the community composition among plant species can reflect the host phylogeny, a phenomenon known as phylosymbiosis. The term “phylosymbiosis” was first coined by Seth Bordenstein and his team, referring to the pattern where the evolutionary relationships of host species are mirrored by the composition of their associated microbial communities [8, 9]. Phylosymbiosis has been observed in wild and domesticated populations, including plants [9–14]. Given these associations, phylosymbiosis provides a potential means for predicting microbial compositions based on host phylogenetic information (Fig. 1a).

In this study, we investigate whether the composition of plant-associated microbiomes can be predicted solely based on the evolutionary relationships of their hosts. To this end, we employed machine learning (ML) models, which are able to capture complex nonlinear patterns inherent to high-dimensional data. Although they have proven success in other domains, their application in computational microbiome research remains relatively sparse [15]. To date, only a few studies have focused on microbial feature classification, such as determining the abundance, diversity, or distribution of microbiota [15, 16]. Additionally, some studies have attempted to predict microbial community composition in response to changes in external features, such as phenotype and environmental variables [15, 17, 18]. For example, Garcia-Jimenez and colleagues (2021) utilized deep learning techniques, specifically autoencoders, for latent feature representation, enabling the prediction of hundreds of taxa, in contrast to the highly aggregated 24 taxa families predicted by Larsen et al. (2012) [15, 17]. Similarly, Ladau et al. (2018) analysed the role of environmental variables in shaping microbial communities as a consequence of climate change, focusing on predicting shifts in microbial diversity rather than their compositions [18]. Recent studies have focused on predicting changes in microbiome compositions in response to ecological perturbations or variations in species assemblages, i.e., the presence/absence of microbiota [19, 20]. Both studies underscore the value of knowing which species are present or absent as a foundation for predicting more complex ecological characteristics, such as species abundances or community compositions circumventing the need for detailed ecological or interaction models. While Asher and Bashan (2024) proposes a”model-free” approach that uses a k-nearest-neighbours regression algorithm to predict species’ abundances based on their presence/absence configuration Michel-Meta and colleagues (2022) developed a deep learning framework that automatically learns the relationship between species assemblages and community compositions. Asher and Bashan (2024) achieved superior performance using a simple KNN approach to predict the abundance of microorganisms compared to their neural network model, across both simulated and real human-associated microbiome data [19]. Although those models have demonstrated the potential to predict microbial compositions, they have notable shortcomings, such as their reliance on complex predictors that may limit generalizability, or their dependence on prior presence/absence information to predict abundance, as well as the computational and data demands required to achieve reliable performance.

The main contributions of this work are as follows:

1. We introduce three customized ML algorithms for predicting microbial abundance using phylogenetic distance metrics derived from aligned Internal Transcribed Spacer (ITS) sequences of host plants (e.g., Hamming distance) as input. Specifically, we propose Hamming Distance-based k-Nearest Neighbour (HD-KNN), Gaussian Process Regression (HD-GPR), and Support Vector Regression (HD-SVR).
2. We develop a comprehensive pipeline that includes data pre-processing, statistical analysis, ML modelling, and model evaluation. This pipeline is generalizable and can be applied to predict the microbiome composition of other plant.
3. We apply the proposed methodology to a real-world dataset comprising 61 plant species with their associated microbiomes and conduct an in-depth post hoc analysis to better understand the conditions under which the best performing model succeeds or fails, offering insights into its performance across different contexts.

## Methodology

### Model overview and notation

Here, we present three customized machine-learning (ML) algorithms that incorporate sequence-based evolutionary information based on the Hamming distance: Hamming Distance-based k-Nearest Neighbour (HD-KNN), Hamming Distance-based Gaussian Process Regression (HD-GPR) and Hamming Distance-based Support Vector Regression (HD-SVR). Furthermore, we define the evaluation metrics for assessing the model performance, which also serve as basis for our downstream analyses. Before introducing the individual ML algorithm, we establish the general notation used throughout our paper. Let $\mathcal{X} \subseteq {\mathbb{R}}^{p}$ be the $p$-dimensional input feature space, and $\subseteq {\mathbb{R}}^{m}$ denote the $m$-dimensional output space, where each output corresponds to a distinct microbial taxon (OTU). We present the input as a random vector $X=\left({X}_{1}, \dots , {X}_{p}\right)\in \mathcal{X}$, where ${X}_{j}$ refers to the $j$-th feature and outputs as a random vector $Y=\left({Y}_{1}, \dots , {Y}_{m}\right)\in \mathcal{Y}$, where ${Y}_{k}$ refers to the abundance of the $k$-th microbial taxon. Drawing $n$ independent and identically distributed (i.i.d.) samples from the joint probability distribution ${\mathbb{P}}_{X}{,}_{Y}$, we obtain the dataset $\mathcal{D}={\left\{\left({x}^{\left(i\right)}, {y}^{\left(i\right)}\right)\right\}}_{i=1}^{n}$, where ${x}^{\left(i\right)}= \left({x}_{1}^{(i)}, \dots , {x}_{p}^{(i)}\right)\in {\mathbb{R}}^{p}$ is the feature vector for the $i$-th plant species, and ${y}^{\left(i\right)}= \left({y}_{1}^{(i)}, \dots , {y}_{m}^{(i)}\right)\in {\mathbb{R}}^{m}$ is the corresponding microbiome profile. The goal of supervised ML is to learn underlying relationship between inputs and outputs by suitable functions $f: \mathcal{X}\to \mathcal{Y}$ from the training data $\mathcal{D}$. In practice, we treat each output variable ${y}_{k}$ independently and formulate the problem as a collection of univariate regression tasks. Accordingly, for each microbial taxon $k\in \left\{1,\dots , m\right\},$ we train a separate model ${f}_{k}: \mathcal{X}\to {\mathbb{R}}$, each using the same input features to map the complete microbial community.

### Customized machine learning algorithms

To predict microbial community compositions from host plant DNA, we use internal transcribed spacer (ITS) region, a phylogenetic marker commonly used in plants. We formulate a regression task where the input features are aligned ITS sequences, and the targets are continuous microbial abundance profiles. Given that the sequences are categorical and aligned, we define a pair-wise similarity between sequences using the normalized Hamming distance [21], which naturally reflects evolutionary differences at the nucleotide level. We adapt three well-established learning algorithms, KNN, GPR, and SVR to operate on this sequence-based, distance-defined input space. For all models, hyperparameter optimization was conducted using a nested cross-validation approach, as described in the Model Evaluation section and detailed in Supplementary Table S1.

### Hamming distance-based k-nearest neighbour (HD-KNN)

K-Nearest Neighbour (KNN) is a non-parametric and instance-based intuitive learning method that predicts the outcome for a new instance by averaging the outcomes of its k nearest neighbours in the input space [22]. In our case, the input consists of aligned sequences, which are purely categorical. Accordingly, we replace the conventionally used Euclidean distance with the normalized Hamming distance, which quantifies dissimilarity by counting the number of mismatched positions between two aligned sequences:

1 $$ d_{H} \left( {x^{\left( i \right)} , x^{\left( k \right)} } \right) = \frac{1}{p} . \mathop \sum \limits_{j = 1}^{p} 1\left( {x_{j}^{\left( i \right)} \ne x_{j}^{\left( k \right)} } \right), $$

where, $p$ is the input sequence dimension, and $1\left(\cdot \right)$ is the indicator function. We refer to this adapted model as HD-KNN. For a given test sequence, the microbial abundance is predicted by computing the arithmetic mean of the abundances associated with the k nearest training sequences. KNN offers a non-parametric baseline that makes no assumptions about the functional form of the input–output relationship. Its simplicity and interpretability make it a well suited baseline for biologically grounded tasks. However, its performance highly depends on the choice of $k$. KNN scales poorly to large datasets due to the need for exhaustive pairwise comparisons at prediction time, but it remains effective in small- to medium-sized settings where similar local patterns dominate.

### Hamming distance-based Gaussian process regression (HD-GPR)

Gaussian Process Regression (GPR) is a nonparametric, probabilistic model that defines a distribution over functions and offers uncertainty estimates for predictions [23]. To use GPR with nucleotide sequence, we adapted the kernel function to reflect biological similarity using the normalized Hamming distance from Eq. (1). Before model fitting, we apply a log transformation to the microbial abundance values due to their skewed nature and then centre the transformed values around zero mean, as required by the GPR prior. We then define a custom, sequence-based kernel in the form of a radial basis function (RBF), in which pairwise similarities are computed using the normalized Hamming distance as follows:

2 $$ k\left( {x^{\left( i \right)} , x^{\left( k \right)} } \right) = \exp \left( { - \frac{{d_{H} \left( {x^{\left( i \right)} ,x^{\left( k \right)} } \right) }}{\sigma }} \right) $$

where ${d}_{H}\left(\cdot ,\cdot \right)$ is the normalized Hamming distance of Eq. (1), and σ is a kernel hyperparameter controlling the decay of the similarity with distance. We refer to this adapted model as HD-GPR, enabling GPR to operate in a sequence-informed space without the need for manual feature engineering. The kernel function determines how inputs relate to each other, making GPRs particularly effective for datasets where relationships are best captured through distances in the input space. This property combined with their nonparametric nature, allows GPRs to perform well even when the number of samples is limited but the number of features is high, a setting where traditional regression methods often fail. Although the computational complexity of GPR scales cubically with the number of samples, making it less efficient for very large datasets, it remains highly effective for small- to medium-sized datasets where capturing complex patterns is essential. In this work, we apply GPRs to a regression task, leveraging its ability to model complex input–output relationships in a high-dimensional feature space.

### Hamming distance-based support vector regression (HD-SVR)

Support Vector Regression (SVR) is a kernel-based method that constructs a margin around the predicted values while penalizing deviations outside an ϵ-insensitive zone [24]. Similar as for HD-GPR, we adapt SVR for sequence data by employing the Hamming distance-based exponential kernel, which we introduced in HD-GPR (Eq. 2) based on the normalized Hamming distance of Eq. (1). The HD-SVR model is trained using the specified kernel matrix and log-transformed microbial abundance values. We refer to this approach as HD-SVR. SVR is particularly suited for learning robust predictive models with high generalization performance under limited data conditions. Its reliance on a sparse set of support vectors makes it more memory-efficient than other kernel methods such as GPR, and allows it to scale better in high-dimensional input spaces. However, the performance of SVR can be more sensitive to kernel choice and associated hyperparameters.

### Evaluation metrics

To assess the performance of our predictive models, we compared the predicted microbial community compositions to the observed ground truth compositions for each plant species. To capture complementary aspects of predictive accuracy, we employed both distribution-based and error-based evaluation metrics. Specifically, we used the Jensen-Shannon Divergence (JSD) to evaluate predictions on a community-wide (species-level) basis across all microbes, and mean and median absolute errors to assess prediction accuracy at the level of individual microbial taxon.

### Jensen-Shannon divergence (JSD)

Jensen-Shannon divergence (JSD) is a symmetrized and smoothed version of the Kullback–Leibler divergence that measures the similarity between two probability distributions [25]. It is particularly well suited for compositional microbiome data due to its bounded nature and sensitivity to differences in both high- and low abundance taxa. In our context, JSD is used to compare the overall predicted and observed microbial compositions at the community level for each plant species. Given two probability distributions $P$ and $Q$, JSD is defined as:

3 $$ JSD\left( {P\parallel Q} \right) = \frac{1}{2}D_{KL} \left( {P\parallel M} \right) + \frac{1}{2}D_{KL} \left( {Q\parallel M} \right), $$

where $M=\frac{1}{2}(P+Q)$ is the average distribution, and D_KL_ denotes the Kullback–Leibler divergence:

4 $$ D_{KL} \left( {P\parallel Q} \right) = \mathop \sum \limits_{i} P\left( i \right) \log \left( {\frac{P\left( i \right)}{{Q\left( i \right)}}} \right). $$

Here, $i$ indexes the microbial taxa of each member of the microbial community. JSD values lie in the range $[0, 1]$, with $0$ indicating identical distributions and $1$ representing maximal divergence. It is widely used in microbiome research for its ability to highlight ecologically meaningful shifts in community structure, though it may also amplify variation in rare taxa.

### Mean absolute error (MAE) and median absolute error (MedAE)

To evaluate model performance at the individual microbial taxon level, we used absolute error metrics. The mean absolute error (MAE) quantifies the average absolute prediction error across all $n$ (or a subset of) plant species for the predictions $\widehat{y}$ of one microbe [26].

5 $$ MAE = \frac{1}{n} \mathop \sum \limits_{i = 1}^{n} \left| {y^{\left( i \right)} - \hat{y}^{\left( i \right)} } \right|, $$

where ${y}^{\left(i\right)}$ and ${\widehat{y}}^{\left(i\right)}$ represent the observed and predicted relative abundances, respectively. MAE provides an interpretable measure of average prediction accuracy. To reduce the influence of large errors or outliers, we also computed the median absolute error (MedAE). This metric summarizes the typical prediction error using the median, making it particularly robust in the presence of skewed error distributions:

6 $$ MedAE = median \left( {\left| {y^{\left( i \right)} - \hat{y}^{\left( i \right)} } \right|} \right) $$

Together, these metrics offer a complementary evaluation framework, capturing both the distributional fidelity of the predicted microbial communities and the specific per-taxon prediction accuracy.

### Data and pre-processing

The aim of this study is to predict the average microbial abundance of each OTU across plant species by using only the phylogeny information of the 61 plant species. In the following, we describe the dataset, the applied data processing pipeline, the predictive modelling approach, and the performance evaluation used to compare our proposed machine learning algorithms. An overview of the entire approach is illustrated in Fig. 1b. The code and data used to reproduce the results of this manuscript are publicly available at https://github.com/JuliaHerbinger/microbiome_prediction.

### Phylogenetic feature space

The internal transcribed spacer (ITS) regions of the nuclear ribosomal DNA from the selected plant species were retrieved from NCBI and are available in the project repository (https://github.com/JuliaHerbinger/microbiome_prediction/tree/main/data). ITS was selected because it was available for all 61 plant species included in the prediction framework and provides sufficient sequence variation to distinguish closely related seed plant species. ITS and ITS2 have been widely used in plant DNA barcoding and species-level discrimination, whereas highly conserved ribosomal markers such as 18S are better suited for broad phylogenetic placement but provide limited resolution among closely related species [27–29]. The ITS sequences were aligned using the Multiple Sequence Comparison by Log-Expectation (MUSCLE) algorithm [30], and the aligned sequences were used to construct the host feature space for the predictive modelling approach. Pairwise normalized Hamming distances were calculated between aligned ITS sequences and used as the primary input to the distance-based models (Eq. 1). Hamming distance was calculated as the number of nucleotide mismatches, including alignment gaps, divided by the total alignment length. We refer to this matrix as plant nuclear ITS-derived host relatedness. This matrix was used as an operational marker-based proxy for host genetic relatedness rather than as a fully resolved, time-calibrated plant phylogeny.

### Microbial target space

The prediction target was the seed-associated bacterial microbiome. The 16S rRNA (V4) data were derived from the Seed Microbiota Database, from the previously published meta-analysis [31]. Because the data were compiled from public studies, sampling period, geographic origin, seed handling, and surface-sterilization procedures were not fully harmonized across all samples. Therefore, we interpret the profiles as seed-associated bacterial communities rather than strictly seed endophytes or epiphytes. Amplicon sequence variant (ASV) counts were normalized using cumulative sum scaling (CSS) with log transformation as implemented in the metagenomeSeq package [32]. The normalized OTU table comprised 11,739 OTUs across 1531 samples from multiple studies. Of these, 1151 samples from 61 plant species with available ITS-derived sequence data were retained for the prediction framework; the remaining 380 samples, from species lacking ITS sequences required to compute host-relatedness features, were excluded from model training and evaluation. The number of seed samples contributing to each species profile varied among host species, reflecting the multi-study origin of the dataset. To focus the prediction task on OTUs with reproducible within-species abundance patterns, we applied a coefficient of variation (CV) filter. For each OTU and each of the 61 plant species, the intra-species CV, calculated as SD divided by the mean across biological replicates within a species, was computed from CSS-normalised values. An OTU was retained if its intra-species CV < 0.5 in at least one plant species. This criterion retains OTUs that are measurably reproducible in at least one host context, while excluding OTUs whose within-species abundance is dominated by replicate noise in every host examined. The threshold of 0.5 represents a conservative balance between removal of high-noise OTUs and preservation of ecologically relevant taxa; its robustness to threshold choice is assessed in Supplementary Results (Supplementary Tables S5, S7; Supplementary Fig. S2a, c). This filtering yielded 372 retained OTUs, representing 3.2% of total OTU richness but accounting for 64.58% of total raw sequencing reads across all 1,151 samples (Supplementary Table S6). Species-level microbiome profiles were obtained by averaging normalised OTU abundances across all samples from the same plant species, yielding a 61-species × 372-OTU target matrix (Y). Abundance coverage was calculated from raw read counts prior to CSS normalisation to reflect the fraction of sequencing depth represented by the retained OTUs.

### Predictive modelling approach

To investigate whether the plant microbiome can be predicted based on phylogeny, we framed the task as a series of univariate regression problems. Each problem focuses on predicting the abundance of a single OTU based on the phylogenetic aligned sequences of the plant species. This approach allows to learn different feature relationships for different OTUs. We compare the three proposed learning algorithms, i.e., HD-KNN, HD-GPR, and HD-SVR, which all leverage HD distances in the feature space for prediction. For HD-KNN, the Hamming distance is used directly to define neighbourhoods, for GPR and the SVR, the same distance is used to construct the kernels as defined in Eq. (2). This consistent use of the biologically meaningful sequence distance ensures comparability across models and overcome the limitation of conventionally used kernels such as radial basis function (RBF), which assumes continuous Euclidian input features and therefore is not suited for categorical sequence data. To put it in a nutshell, our goal is to identify robust machine learning models for the prediction of individual microbial taxa by systematically comparing the three proposed sequence informed learning algorithms, each leveraging the same phylogenetic input.

### Model evaluation

To provide a fair comparison between the three learning algorithms, we perform a nested cross-validation (Fig. 1b) where hyperparameter optimization (HPO) is conducted in the inner resampling using a fivefold cross-validation. On each outer fold, we compare the three different learning algorithms based on the best found hyperparameter configurations. We apply the leave-one-out strategy to evaluate the model performance on the outer folds. For each OTU, we apply the following procedure to train and evaluate the learning algorithms: One of the 61 species is left out as a test case. We then perform a fivefold cross-validation within the remaining 60 species to conduct HPO, finding well-performing hyperparameter configurations for each of the three learning algorithms. Specifically, for HD-KNN, we tune the number of neighbour’s k using a grid search over eight evenly spaced values; for HD-GPR, we perform a grid search over 10 evenly spaced values for the sigma parameter; and for HD-SVR, we use random search with 10 evaluations over the parameters C and sigma. The full details of these hyperparameter search strategies, including search ranges and evaluation counts, are provided in Supplementary Table S1. After identifying the best hyperparameters, we fit each learning algorithm on the 60 remaining training species and compare their performance on the left-out species. This procedure is repeated for each of the 61 species and for each OTU. The leave-one-out approach was chosen because the sample size is small, and while some families are well represented by various species, the dataset also includes species with few or no closely related species phylogenetically. Thus, we aim to understand whether we can predict the average microbial abundance of a new species knowing only its phylogenetic sequence information based on our existing database. This nested cross-validation setup ensures robust model evaluation and a fair comparison by separating model tuning from performance estimation, thereby reducing the risk of overfitting. To assess whether model performance exceeded simple baseline expectations, we implemented additional baseline and null comparisons using the same leave-one-species-out evaluation protocol and per-species JSD metric as the main benchmark. The global mean baseline predicted each left-out species as the mean 372-OTU profile of the remaining training species, while the all-neighbour KNN baseline used all 60 training species as neighbours and is therefore equivalent to simple averaging. The family/group mean baseline predicted each left-out species from the mean microbiome profile of training species within the same predefined host group, where available. To test whether the Hamming-distance representation itself added predictive value, we also included a Euclidean KNN baseline using fixed k = 5 and Euclidean distance on the same numerical ITS alignment features. Finally, we implemented a permuted HD-KNN null model using fixed k = 5, in which the correspondence between plant species and ITS-derived feature vectors was randomly disrupted across 100 permutations, thereby breaking the biological link between host identity and microbiome profiles.

### Statistical analysis

To assess whether host relatedness among plant species was associated with seed-associated bacterial community composition, we applied Mantel tests correlating pairwise plant nuclear ITS-derived host-relatedness matrices with pairwise microbial community dissimilarity matrices. Mantel tests were performed across all 61 species and separately for Brassicaceae, Poaceae, and the remaining species. Both Pearson and Spearman correlation coefficients were computed using the *mantel* function from the vegan R package [33] with 1000 permutations and no multiple-testing correction was applied.

## Results

### Phylosymbiosis in seed microbiomes

**Fig. 2:**
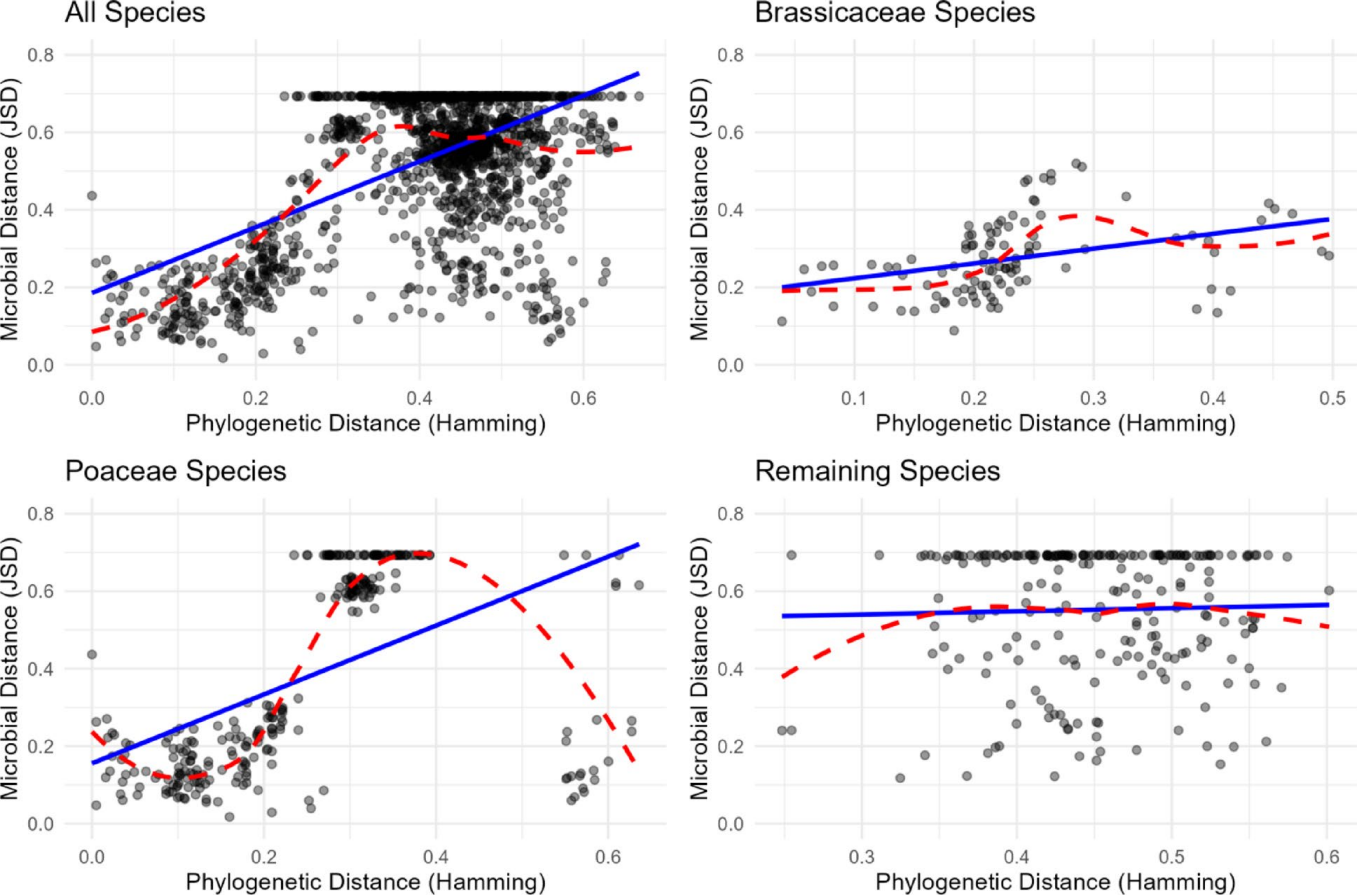
Pairwise plant nuclear ITS-derived host relatedness, measured as normalised Hamming distance from ITS sequences, versus microbial community dissimilarity, measured as Jensen-Shannon divergence (JSD), for all species combined (top left), Brassicaceae (top right), Poaceae (bottom left), and the remaining species (bottom right). Each point represents one pairwise combination of plant species. The solid blue line shows the ordinary least-squares (OLS) linear regression fit. The dashed red curve shows a LOESS (locally estimated scatterplot smoothing) fit using a span of 0.75, included to reveal potential non-linear trends in the distance-dissimilarity relationship. Mantel test statistics for each panel are reported in Table 1

To assess whether phylogenetic relatedness among plant species is reflected in the composition of their seed microbiomes, we performed Mantel tests correlating pairwise phylogenetic distances derived from normalized Hamming distances of aligned ITS sequences with microbial community dissimilarities, measured by JSD. Across all 61 species, we found a significant correlation between plant phylogeny and microbiome similarity, indicating a general phylosymbiotic pattern (Table 1; Fig. 2). At the global scale (all 61 species), the Pearson correlation (r = 0.561) was stronger than the Spearman correlation (r = 0.268), suggesting a partial linear component in the host-microbiome distance relationship when species from diverse families are considered together. However, this pattern reversed within individual family groups, where Spearman correlations were equal to or higher than Pearson correlations (*Brassicaceae*: 0.498 vs. 0.361; *Poaceae*: 0.629 vs. 0.509), indicating a monotonic rather than strictly linear relationship at finer taxonomic scales. In contrast, no significant correlation was detected for the remaining species, likely due to low sample size and sparse phylogenetic representation. Figure 2 illustrates an overall positive trend both across all species and within the two major families. However, the relationships are not strictly linear nor purely monotonic. These descriptive observations underscores the need for flexible, nonparametric ML models capable of capturing such complex, nonlinear relationships.

**Table 1:** Mantel test results indicating the correlation between plant phylogenetic distance (normalized Hamming distance from ITS sequences) and microbial community dissimilarity (Jensen-Shannon divergence)

Method	All	*Brassicaceae*	*Poaceae*	Remaining species
Pearson	0.561 (*p* = 0.001)	0.361 (*p* = 0.063)	0.509 (*p* = 0.001)	0.03 (*p* = 0.34)
Spearman	0.268 (*p* = 0.001)	0.498 (*p* = 0.002)	0.629 (*p* = 0.001)	0.003 (*p* = 0.48)

Both Pearson and Spearman correlation coefficients are reported, with associated *p*-values. Analyses were conducted across all 61 species and separately for the Brassicaceae, Poaceae, and remaining species (i.e., those not belonging to either family)

### Model performance across plant groups

We evaluated the performance of our three proposed ML models; HD-KNN, HD-GPR, and HD-SVR in predicting microbial community compositions from plant phylogenetic distances. Model accuracy was assessed by the JSD between predicted and observed OTU profiles. Across all 61 plant species, HD-KNN achieved the best average performance (JSD = 0.276 ± 0.171), followed closely by HD-GPR (0.281 ± 0.180) and HD-SVR (0.299 ± 0.193) (Table 2). The performance advantage of HD-KNN was most pronounced within the *Poaceae* and *Brassicaceae* families, where it yielded a mean JSD of 0.176 ± 0.100, compared to 0.188 ± 0.116 for HD-GPR and 0.199 ± 0.137 for HD-SVR (Table 2). However, among species outside these two families, HD-GPR slightly outperformed HD-KNN, suggesting that the relative effectiveness of each model may vary with phylogenetic context.

**Table 2:** Arithmetic mean ± standard deviation of Jensen-Shannon divergence (JSD) for each model

	Mean ± SD of JSD		
Model	All Species	*Brassicaceae* & *Poaceae*	Remaining Species
HD-KNN	**0.276 ± 0.171**	**0.176 ± 0.100**	0.452 ± 0.122
HD-GPR	0.281 ± 0.180	0.188 ± 0.116	**0.444 ± 0.157**
HD-SVR	0.299 ± 0.193	0.199 ± 0.137	0.476 ± 0.147

Results are shown for all species, for the combined group of Brassicaceae and Poaceae, and for species not belonging to these two families. Bolded values indicate the best average performance within each group

The 61 species-level microbiome profiles were generated from 1151 seed-associated bacterial samples, with sample numbers varying among host species because the dataset integrates multiple independent studies (Supplementary Table S19). Because host representation was uneven across plant groups, we assessed the influence of dense *Oryza* sampling on prediction performance. Of the 61 plant species analysed, *Poaceae* comprised 24 species, of which 18 belonged to *Oryza*, representing 75% of *Poaceae* and 29.5% of the full dataset. Prediction accuracy was strongest in densely sampled clades, particularly *Oryza*-rich *Poaceae* and the *Brassicaceae* group. Mean HD-KNN JSD was 0.157 ± 0.123 for *Oryza* species, 0.179 ± 0.124 for *Poaceae* including *Oryza*, and 0.173 ± 0.040 for the *Brassicaceae* group. Excluding *Oryza* increased *Poaceae* JSD from 0.179 to 0.242, indicating weaker prediction accuracy for non-*Oryza Poaceae*. The combined *Brassicaceae* and *Poaceae* group changed from 0.176 to 0.193 after *Oryza* exclusion. This sensitivity analysis is descriptive because the non-*Oryza Poaceae* subgroup contained only six species (Supplementary Tables S16-S18; Supplementary Fig. S3-S5).

To determine whether this performance exceeded simple baseline expectations, we compared the tuned models against five baseline/null comparisons using the same leave-one-species-out evaluation framework (Supplementary Table S8; Supplementary Fig. S3). The global mean baseline, which predicts each left-out species as the average OTU profile of the remaining 60 species, yielded a mean JSD of 0.418 ± 0.094. The all-neighbour KNN baseline was numerically identical to the global mean baseline. In comparison, tuned HD-KNN achieved a substantially lower mean JSD of 0.276 ± 0.171 and outperformed the global mean baseline in 51 of 61 species (83.6%; paired one-sided Wilcoxon test, *p* < 0.001; Supplementary Table S9). HD-KNN also outperformed the family/group mean baseline (0.312 ± 0.134) in 38 of 61 species (62.3%; p = 0.014), indicating that coarse family/group-level structure explains part, but not all, of the predictive signal. HD-KNN also achieved lower mean JSD than Euclidean KNN (0.302 ± 0.207; *p* = 0.032), although the effect size was modest (ΔJSD = 0.026). Finally, all 100 permuted HD-KNN runs yielded higher mean JSD than the observed HD-KNN model (null mean JSD = 0.456; conservative empirical *p* = 0.0099), supporting that the observed performance was not simply an artefact of species-level averaging or the filtered target space.

### Variation in predictive accuracy

**Fig. 3:**
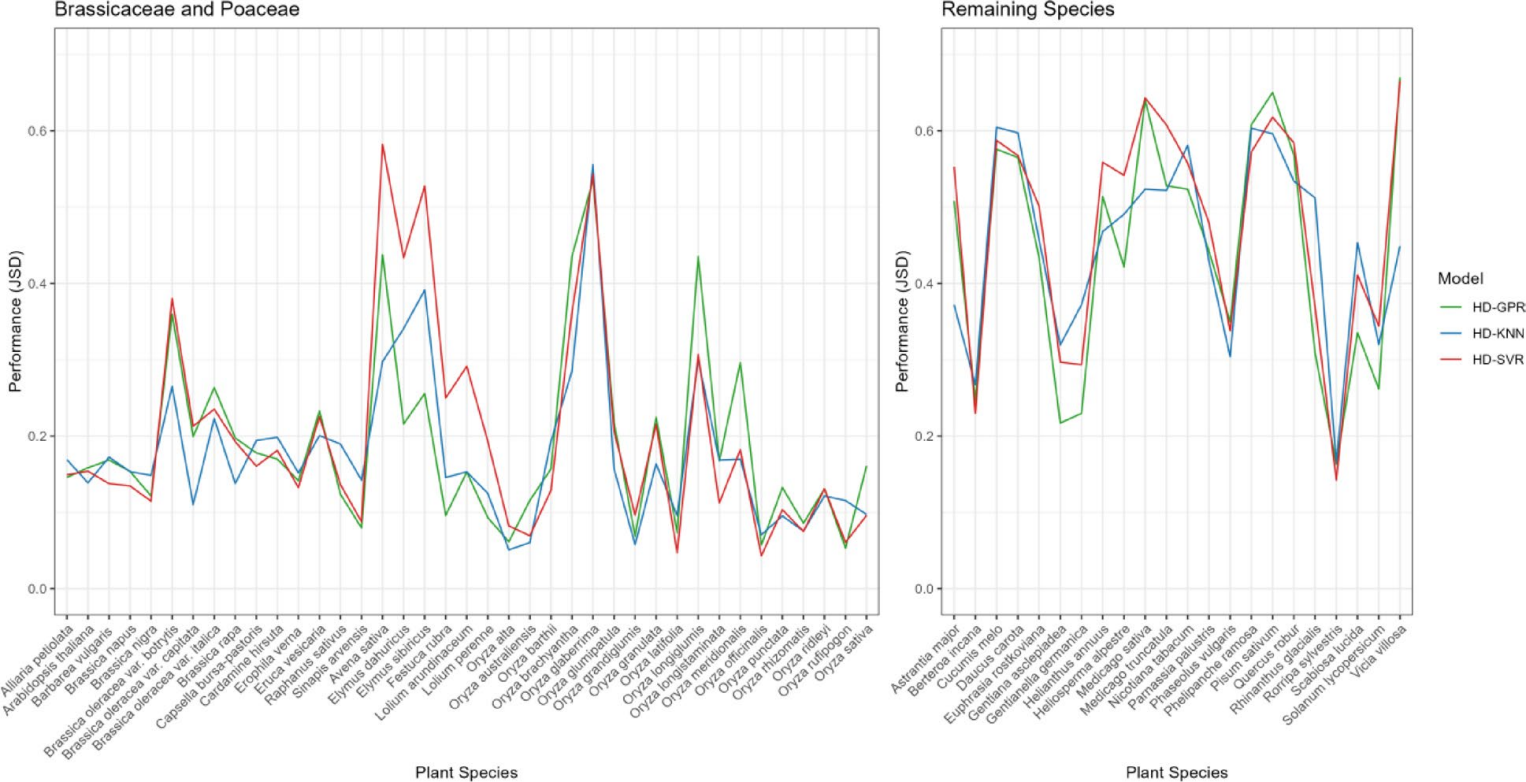
Jensen-Shannon divergence (JSD) for each species under three machine learning models. The left panel shows species from Brassicaceae and Poaceae; the right panel shows all remaining species. HD-KNN typically outperforms others in densely clustered clades, while HD-GPR shows stronger performance for phylogenetically isolated species

To gain a more detailed understanding of the model performance beyond group averages, we examined the prediction error (JSD) for each held-out species (Fig. 3). Among species belonging to *Brassicaceae* and *Poaceae*, HD-KNN tended to yield lower prediction errors compared to the other models, particularly for taxa with closely related neighbours. Its strong performance likely stems from its local modelling strategy, which benefits from the presence of nearby relatives especially in densely clustered families (Supplementary Fig. 1). In contrast, several phylogenetically isolated species (e.g., *Rhinanthus glacialis, Gentianella germanica, Gentiana asclepiadea*) were better predicted by HD-GPR, likely due to its ability to model complex, non-local patterns. For these species, the JSD exhibits greater sensitivity to the number of neighbours, indicating that the relationship between input features and microbial targets might be based on more global patterns, making HD-GPR a more suitable modelling approach. Species within the “Remaining” group tended to show higher JSD overall, with performance varying widely across models. HD-SVR was generally less accurate than HD-KNN or HD-GPR, especially for species within clustered families. Notably, HD-SVR struggled with species such as *Avena sativa, Elymus dahuricus, Elymus sibiricus*, which exhibited rapidly increasing JSD values as more distant neighbours are included. This indicates that prediction accuracy relies mainly on the closest neighbours. Species that were consistently the most difficult to predict regardless of the model tended to be phylogenetically distant from all others and often exhibit decreasing JSD with increasing neighbourhood size.

**Fig. 4:**
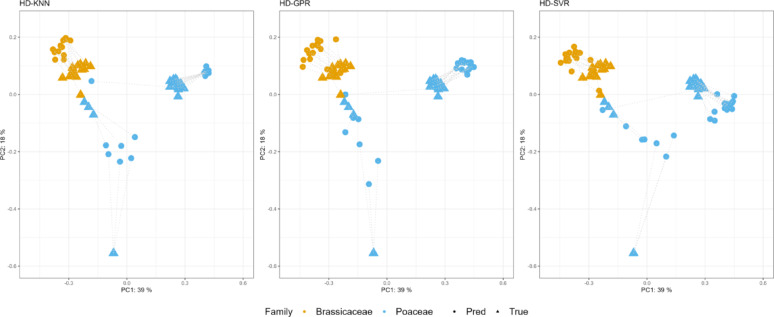
The figure illustrates the true microbiome composition compared to the predictions of each model mapped to the first two principal components of a PCoA based on the JSD. The PCoA is calculated and visualized for species within the two biggest families (Brassicaceae and Poaceae), where the color-coding indicates to which family each species belongs, while the shape of the sign indicates if it represents the prediction or true label. The dotted lines show which true label belongs to which prediction

Principal Coordinates Analysis (PCoA) based on the JSD between predicted and actual profiles showed that predictions from HD-KNN and HD-GPR closely approximated the true microbial compositions, which can be observed in Fig. 4. The ordinations for HD-KNN and HD-GPR revealed broadly similar patterns, with predicted points mapping relatively close to their true counterparts for many species. Notably, HD-KNN exhibited lower variability within the two major families, *Brassicaceae* and *Poaceae*, whereas HD-GPR produced shorter distances for several outlier species aligning with its superior performance in capturing more complex or globally varying relationships.

### Effect of phylogenetic neighbourhood size (k) on HD-KNN accuracy

**Fig. 5:**
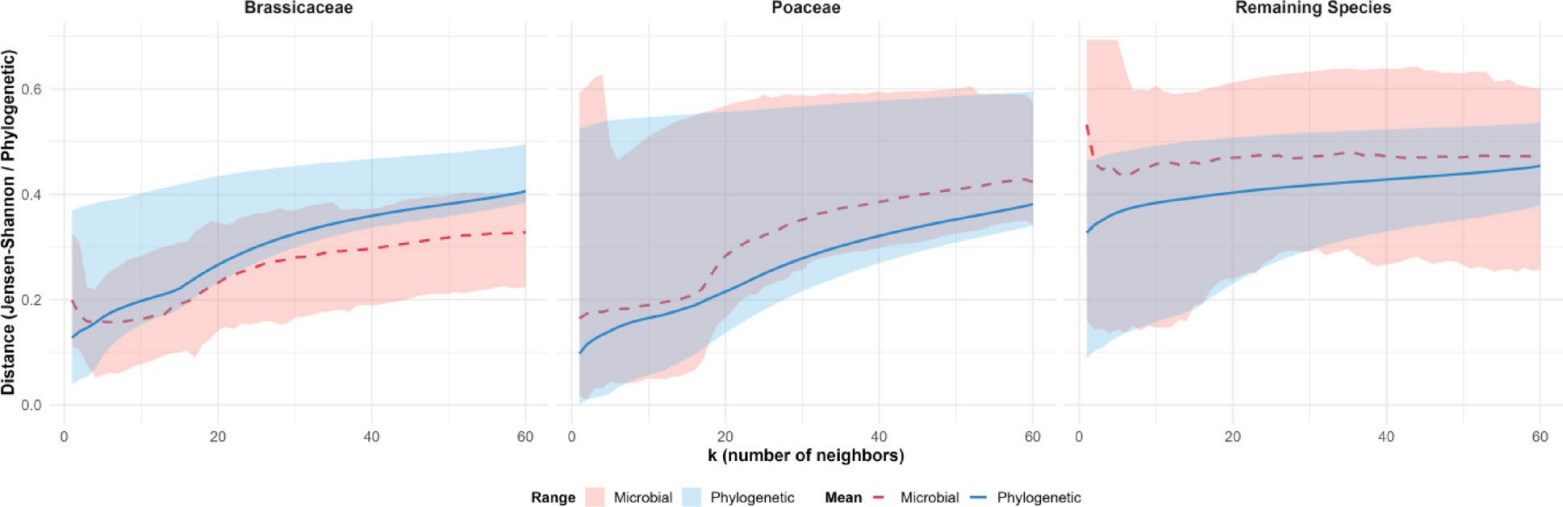
The plots show how predictive performance of the HD-KNN model varies with the number of neighbours (k) for Brassicaceae, Poaceae, and remaining species. The solid lines represent the GroupWise mean microbial (dashed red) and phylogenetic (solid blue) distances, while the shaded areas indicate the full range (min–max) of observed distances

To investigate the impact of the neighbourhood size on the predictive performance in HD-KNN, we compute the average JSD between predicted and observed microbiome profiles for each species across a range of k values (Fig. 5). Species within *Poaceae* and *Brassicaceae* exhibit the lowest prediction errors for smaller values of k, with optimal performance typically achieved when using 1–4 neighbours (Supplementary Table S3). For these families, increasing k lead to progressively higher JSD values, indicating reduced prediction accuracy, as phylogenetically distant species were included. This trend was confirmed by plotting group-wise JSD against k for species within and outside the two major families. In *Brassicaceae* and *Poaceae*, both microbial and phylogenetic distances increased with k, and the lowest JSD values were obtained using small neighbourhoods (Fig. 5). In contrast, species outside these families displayed no consistent improvement or degradation in performance with increasing k, and their average JSD values remained high across all neighbourhood sizes (Supplementary Table S3; Fig. 5). These results suggest that HD-KNN performs best when phylogenetically similar neighbours are available and its accuracy declines when such local information is diluted by the inclusion of distant species.

### Taxon-level prediction difficulty across species

**Fig. 6:**
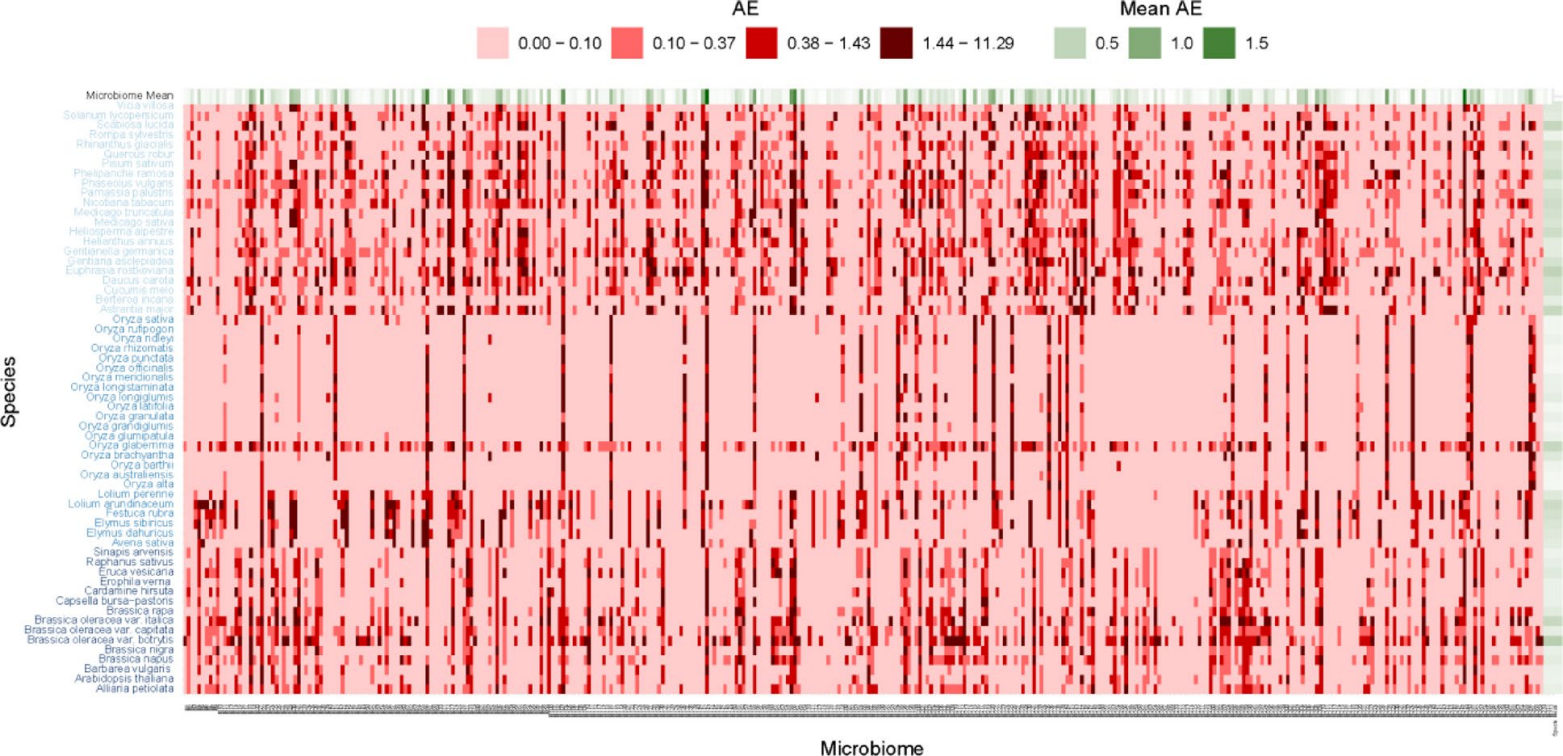
Heatmap showing the absolute deviation between the predicted and true microbial abundance for the tuned HD-KNN model, shown for each hold-out species and each OTU (microbiome). The red colour scale indicates the magnitude of error, ranging from light (small errors) to dark (high errors). Specifically, the intervals are defined as follows: 0.00–0.10 corresponds to the minimum up to the 75th percentile of errors, 0.10–0.37 to the 75th-85th percentiles, 0.38–1.43 to the 85th-95th percentiles, and values above 1.44 represent the top 5% of errors. Green shading in the top row and last column indicates the mean absolute error (MAE) at the microbiome and species levels, respectively. The species names are color-coded in blue according to family affiliation: dark blue for Brassicaceae, medium blue for Poaceae, and light blue for species not belonging to either of these two families

To assess variation in prediction accuracy across both plant species and microbial taxa, we computed the absolute deviation between predicted and observed OTU abundances using the optimized HD-KNN model (Fig. 6). Species within *Brassicaceae* and *Poaceae* generally exhibited lower overall prediction errors compared to other species. Within these families, certain OTUs were consistently well predicted, while others showed high prediction error across all species groups. This taxon-specific variation was particularly evident in *Poaceae*, where several OTUs yielded near-zero error for all member species, in contrast to others that remained difficult to predict regardless of phylogenetic proximity. To identify the most challenging taxa, we ranked OTUs by their median absolute error (MedAE) across all plant species. The OTUs with the highest MedAE across all species were identified, with taxon M143 (*Pseudomonas*) ranking highest overall and also appearing among the least predictable taxa in *Poaceae* and the remaining species group. The top 10 most difficult-to-predict OTUs within each group (All species, *Brassicaceae*, *Poaceae*, remaining), are listed in Supplementary Table S2. To better characterize the filtered target space used for prediction, we compared the 372 retained OTUs with the 11,367 discarded OTUs. The retained set accounted for 64.58% of total raw sequencing reads despite representing only 3.2% of OTU richness, whereas the discarded OTUs accounted for 35.42% of reads (Supplementary Table S6; Supplementary Fig. S2b). The retained set was dominated by *Proteobacteria, Actinobacteria,* and *Firmicutes*, and the most frequent classified retained genera included *Sphingomonas, Pseudomonas, Bacillus,* and *Methylobacterium* (Supplementary Tables S10-S11). No OTU met a strict core criterion (> 0.1% relative abundance in ≥ 80% of samples), so the retained set should not be interpreted as core-enriched (Supplementary Table S13). Among the 20 highest-error OTUs within the retained benchmarked target space, 6 were assigned to *Pseudomonas*, 2 to *Pantoea*, and 4 to *Enterobacteriaceae*, indicating that abundant generalist taxa remained difficult to predict from plant ITS-derived host relatedness alone (Supplementary Tables S15).

## Discussion

In this study we introduced three customized ML models that leverage the concept of phylosymbiosis to predict the seed bacterial microbiome composition. Distinct from previous prediction approaches [19], our framework relies exclusively on host phylogenetic distances as input, requiring no prior microbial information. Our results demonstrate that, among the proposed models, the HD-KNN algorithm achieved the highest average predictive performance, especially within phylogenetically dense clades where phylosymbiotic patterns have been detected. These findings highlight the potential for using low-input predictors such as host phylogeny to predict the identity and composition of the microbiome.

While we detected phylosymbiotic signals across the 61 investigated plant species, phylosymbiosis was more evident within *Poaceae* and *Brassicaceae* i.e., the two families in the dataset with highest number of species. Species outside these clades showed little to no phylosymbiotic signal, which in turn resulted in low prediction accuracy. This discrepancy is not surprising since *Poaceae* and *Brassicaceae* had 24 and 15 species respectively, while the number of species in the remaining families were between 1 and 5 species/family. It has been documented that for detecting phylosymbiotic patterns, it is important to have sufficient phylogenetic resolution or higher number of samples within clades [9]. Despite this limitation, our results demonstrate that ITS-derived host relatedness carries partial predictive information for seed bacterial community composition and OTU abundance profiles. Among the evaluated models, the HD-KNN achieved the lowest average JSD, particularly within the two well-represented families. This is likely due to its reliance on local similarity [34], which effectively captures the fine-scale phylogenetic structure present in densely sampled clades. In contrast, the performance of HD-KNN declined when predicting microbiomes of phylogenetically distant and/or isolated species where the nearest neighbour is likely to be from a different clade. Furthermore, for HD-KNN smaller neighbourhood sizes (k = 1–4) often yielded improved predictions. While similar results have been previously reported for human microbiome data [19], the fact that even single phylogenetic neighbours were often sufficient to generate accurate abundance predictions.

By contrast, HD-GPR performed relatively better in phylogenetically sparse regions. This may reflect its ability to capture broader, non-linear patterns across distantly related species through kernel based similarity functions [35] rather than relying on immediate neighbours. These trends suggest that model performance might depend on the phylogenetic density of the training data, with local models such as HD-KNN benefiting from densely sampled clades, and more flexible models like HD-GPR better suited for capturing patterns in more diffuse phylogenetic spaces. However, given the limited sample size and unbalanced representation across clades, we caution against drawing strong conclusions regarding the optimal choice of the model for different phylogenetic contexts. Future studies incorporating larger and more evenly sampled phylogenies will be essential to further reveal these relationships.

While our nested cross-validation framework ensured rigorous model evaluation, it also introduced substantial computational complexity. Specifically, we used a leave-one-out strategy across 61 species in the outer loop and a fivefold cross-validation in the inner loop to tune hyperparameters for each of the three models, applied separately to 372 OTUs. Although this approach was necessary to obtain reliable estimates in a relatively small dataset, it may become impractical for larger or more complex datasets. Future implementations could benefit from multivariate or deep learning approaches that model multiple OTUs jointly, potentially capturing covariation among microbes and reducing redundancy in computation. Similarly, more efficient hyperparameter tuning strategies could further improve scalability without compromising performance. Beyond overall model performance, our results revealed substantial variation in the predictability of individual microbial taxa. Several OTUs that were consistently among the hardest to predict were not rare or peripheral: 6 Pseudomonas, 2 Pantoea, and 4 Enterobacteriaceae OTUs appeared among the 20 highest-error OTUs across the HD-KNN benchmark. These groups are frequently reported as common seed-associated bacteria [31], yet their abundance patterns were not well captured by plant nuclear ITS-derived host relatedness alone. This may reflect their ecological generalism and flexible transmission routes. Generalist seed-associated bacteria can be introduced through multiple pathways, including vertical transmission, environmental acquisition, seed surface colonisation, dispersal, and post-harvest [36, 37]. Their abundance may therefore depend more strongly on local environmental exposure, nutrient availability, and seed handling than on conserved host-lineage signals. Supporting this interpretation, 91.4% of Pseudomonas OTU richness in the unfiltered table was removed by the CV < 0.5 filter, indicating high within-species variability rather than stable host-specific abundance. In contrast, more predictable taxa such as Methylobacterium may include lineages with more stable host associations [38]. Together, these results suggest that taxon-level predictability from host relatedness may depend not only on abundance or prevalence, but also on transmission mode and ecological strategy. However, because the present meta-analysis cannot distinguish vertically transmitted seed endophytes from horizontally acquired or surface-associated bacteria, this interpretation remains a hypothesis for future compartment-resolved and transmission-tracking studies.

The ability to predict the seed bacterial microbiome from ITS-derived host relatedness suggests potential applications in both ecological and applied contexts. From an ecological perspective, our findings support the notion that host evolutionary history shapes microbiome assembly, particularly in seeds, a key compartment for potential vertical transmission [39, 40]. From an applied standpoint, phylogeny-based models could eventually help prioritise microbiome predictions for unsampled plant species, particularly when closely related reference species are available. Additionally, such predictive tools may guide the design of synthetic microbial consortia by identifying phylogenetically compatible host-microbe combinations, enhancing colonization success and functional outcomes [41]. As microbiome management becomes a key lever in climate-resilient agriculture, low-input approaches like the one proposed here may help integrate evolutionary insights into future plant-microbiome engineering [42]. Several limitations should be considered when interpreting these findings.

Host relatedness was represented using a single plant nuclear ITS marker. ITS was selected because it provides higher species-level resolution than conserved ribosomal markers such as 18S [27] and was available for all 61 host species. However, ITS is a neutral locus with no direct role in plant–microbe interactions. The observed predictive signal should therefore be interpreted as evidence that ITS-derived host relatedness covaries with seed-associated bacterial community composition, not that the ITS locus itself drives microbiome assembly. It is important to note that this pattern can be explained by at least two non-exclusive explanations: direct phylosymbiotic co-evolution [8] and indirect chemical mediation, whereby related hosts share similar seed chemical environments that influence microbial recruitment [31, 39, 43]. A further consideration is that host-relatedness-based prediction may not apply equally to all members of the seed-associated bacterial community. The weak predictability of abundant generalist taxa such as Pseudomonas and Pantoea is consistent with the idea that some dominant seed-associated bacteria may be shaped less by conserved host-lineage signals than by ecological opportunity and local exposure. These genera are frequently reported as common seed-associated bacteria and can occur across diverse plant hosts and environments [31, 36]. They may also reach seeds through multiple transmission routes, including inheritance from parental tissues as well as acquisition from flowers, fruits, soil, or post-harvest environments [36, 37, 44]. Their abundance may therefore depend on local microbial source pools, nutrient availability, seed chemistry, environmental exposure, and study-specific handling procedures.

This does not contradict a host-relatedness signal at the community level. Instead, it suggests that phylogeny-based predictability is likely taxon-dependent. Taxa that are more consistently filtered by host traits, seed structure, or conserved reproductive chemistry may show stronger host-lineage-associated patterns, whereas ecological generalists may show weaker predictability because they respond to multiple and context-dependent colonization pathways. Future studies integrating seed metabolomics, source tracking, compartment-resolved sampling, and host genomic or phylogenomic data will be needed to distinguish vertically structured, host-filtered taxa from environmentally acquired generalists.

Another limitation concerns the OTU filtering step. The prediction framework was evaluated on the 372 OTUs retained after applying the CV < 0.5 reproducibility filter. These OTUs represented only 3.2% of total OTU richness but accounted for 64.58% of total raw sequencing reads. Thus, the current benchmark primarily evaluates prediction of the dominant and more reproducible fraction of the seed-associated bacterial microbiome, rather than the full rare biosphere. The influence of this filtering decision was assessed through threshold sensitivity analysis. Mean HD-KNN JSD changed gradually from 0.2534 to 0.2757 across CV thresholds from 0.2 to 0.5, indicating that the selected threshold did not represent an abrupt or uniquely favourable cutoff. However, because the benchmark was intentionally restricted to reproducible high-abundance OTUs, the current results should be interpreted as demonstrating predictive structure within the dominant and reproducible fraction of the seed-associated bacterial community, rather than across the complete 11,739-OTU community space. Future work should therefore test whether host-distance-based prediction also extends to rare, low-abundance, or highly variable OTUs that were excluded by the reproducibility filter. The uneven representation of host species across plant groups should also be considered. In particular, *Oryza* constituted 18 of 24 *Poaceae* species and 29.5% of the full 61-species dataset. This dense sampling of closely related *Oryza* species likely contributed to the high apparent predictability of *Poaceae*, because the leave-one-species-out framework provided multiple closely related *Oryza* neighbours for each left-out *Oryza* species. After excluding *Oryza*, *Poaceae* prediction accuracy decreased, although this comparison remains descriptive because only six non-*Oryza Poaceae* species were available. These results suggest that host-distance-based prediction benefits from dense sampling of closely related hosts. This is biologically informative, because close relatives may share conserved seed traits, reproductive chemistry, and microbial filters, but it also limits generalisability across sparsely sampled or phylogenetically isolated lineages. Importantly, because HD-KNN relies on local nearest-neighbour relationships rather than global host-group frequencies, dense *Oryza* sampling is expected to mainly affect predictions within the *Oryza*-rich *Poaceae* region rather than predictions for phylogenetically distinct host groups. Future studies should therefore use more balanced host representation across plant families and genera to determine how well the framework performs beyond densely sampled clades.

More broadly, while our findings demonstrate host relatedness can predict part of the seed bacterial microbiome, the study is limited to one plant compartment, one microbial group, and a modest number of species with uneven clade representation. Moreover, environmental, functional, and trait-based data were not included but could enhance model resolution and biological interpretability. Future work should expand to other microbial kingdoms, compartments, and ecological variables to better capture the complexity of host-microbiome interactions. Despite these limitations, our results offer a robust foundation for predictive microbiome modelling and open new avenues for integrating evolutionary context into microbiome-informed agriculture and ecological forecasting.

## Conclusion

This study demonstrates that seed-associated bacterial microbiomes can be predicted by machine learning models based on plant phylogenetic information alone. Phylogenetically close plant species tend to harbour more similar seed microbiomes, supporting the concept of phylosymbiosis and potential microbial inheritance. Among the models tested, HD-KNN was the most effective in phylogenetically dense clades, while HD-GPR offered broader generalization across sparser regions. These results suggest that host-relatedness-based models could help prioritise microbiome predictions for unsampled plant species, particularly when closely related reference species are available. Potential applications in crop breeding or microbiome-informed conservation remain exploratory, but such frameworks could eventually help identify host lineages expected to harbour similar seed-associated bacterial communities or recurrent microbial groups of agronomic interest. However, these applications will require validation in compartment-resolved datasets that integrate environmental, ecological, metabolomic, functional, and host trait information. Extending the framework to other plant compartments and microbial kingdoms, including fungi, represents an important direction for future research.

## Supplementary Information

Below is the link to the electronic supplementary material.

Supplementary Material 1

Supplementary Material 2

## Data Availability

Data availability Seed microbiota OTU tables and metadata were obtained from the Seed Microbiota Database 37 available at 10.15454/2ANNJM. Internal Transcribed Spacer (ITS) sequences for the 61 plant species used to compute the phylogenetic distances were retrieved from NCBI GenBank. All processed sequence data are included in the GitHub repository associated with this study. Materials availability All materials and datasets used in this study are available via the project GitHub repository (https://github.com/JuliaHerbinger/microbiome_prediction/tree/main/data). Code availability All analysis scripts, processed data, and code for model training, evaluation, and figure generation are freely available on GitHub (https://github.com/JuliaHerbinger/microbiome_prediction).
