# Supplementary material for: Predicting the seed microbiome using phylogeny-driven machine learning
Source: Environ Microbiome. 2026 Jul 13;21:88. doi: 10.1186/s40793-026-00936-1 (PMC13366936; doi:10.1186/s40793-026-00936-1)
Supplement: Supplementary file 2 — Supplementary Material 2 [file 40793_2026_936_MOESM2_ESM.docx]

Phylogeny-based prediction of seed microbiome composition using machine learning

Julia Herbinger^1†^, Dinesh Kumar Ramakrishnan^2,3^^†^, Jannik Reißfelder^1^, Majharulislam Babor^1^, Marina Höhne^1,4^, Ahmed Abdelfattah^2*^

1 Department of Data Science, Leibniz Institute for Agricultural Engineering and Bioeconomy (ATB), Max-Eyth-Allee 100, 14469 Potsdam, Germany

2 Department of Microbiome Biotechnology, Leibniz Institute for Agricultural Engineering and Bioeconomy (ATB), Max-Eyth-Allee 100, 14469 Potsdam, Germany

3 Institute for Biochemistry and Biology, University of Potsdam, Karl-Liebknecht-Str. 24/25, 14476 Potsdam, Germany

4 Department of Computer Science, University of Potsdam, An der Bahn 2, 14476 Potsdam, Germany

† Equally contributing authors

*Corresponding author [aabdelfattah@atb-potsdam.de](mailto:aabdelfattah@atb-potsdam.de)

Julia Herbinger: [julia.herbinger@gmail.com](mailto:julia.herbinger@gmail.com)

Dinesh Kumar Ramakrishnan: 0009-0009-0339-4029 [DRamakrishnan@atb-potsdam.de](mailto:DRamakrishnan@atb-potsdam.de)

Jannik Reißfelder: [jannik.reissfelder@gmail.com](mailto:jannik.reissfelder@gmail.com)

Majharulislam Babor: [MBabor@atb-potsdam.de](mailto:MBabor@atb-potsdam.de)

Marina Höhne: 0000-0003-3090-6279 [hoehne5@uni-potsdam.de](mailto:hoehne5@uni-potsdam.de)

Ahmed Abdelfattah: 0000-0001-6090-7200 [AAbdelfattah@atb-potsdam.de](mailto:AAbdelfattah@atb-potsdam.de)

# **Supplementary Figures**


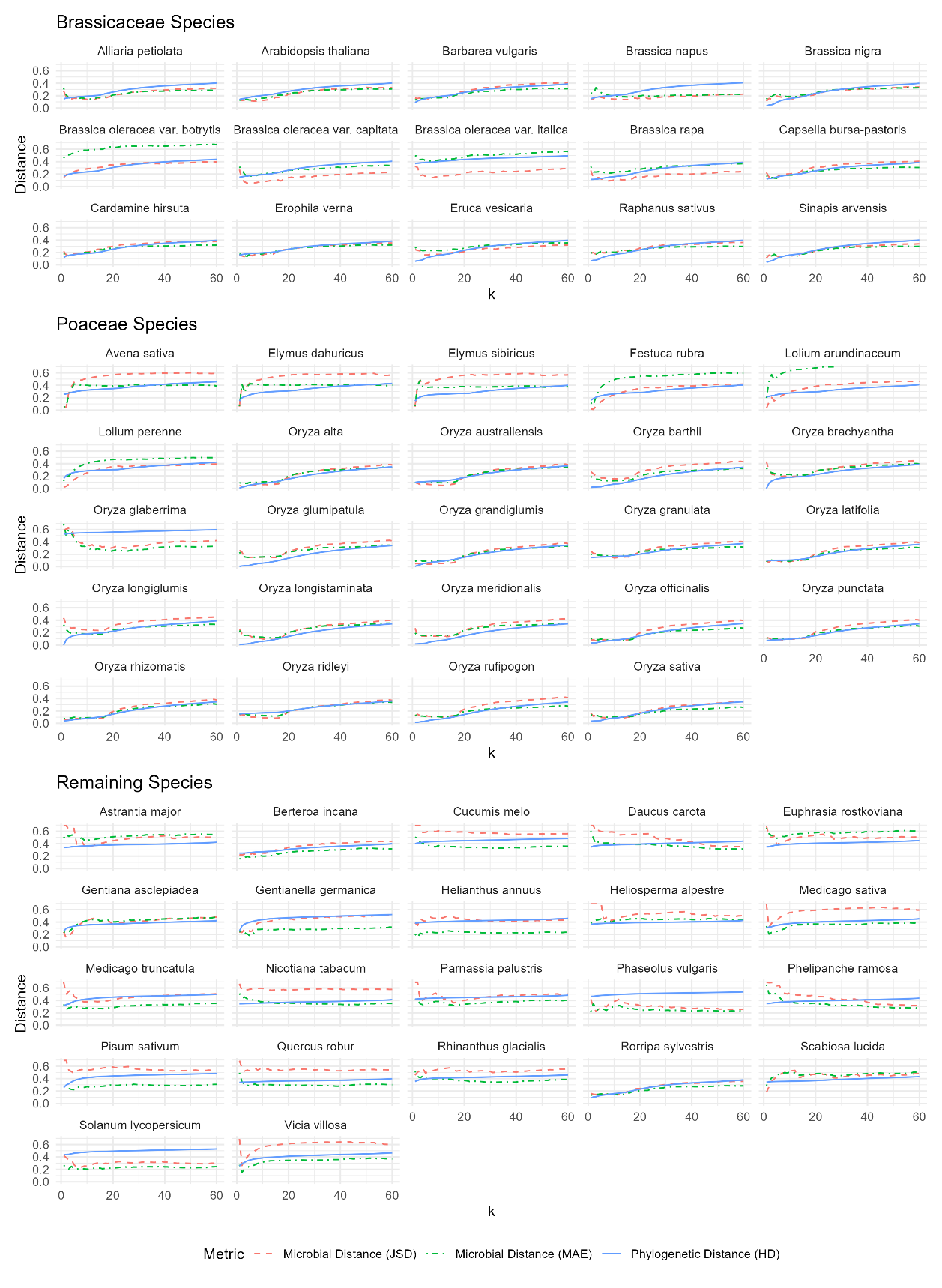
**Supplementary Figure S1. Effect of neighbour number on HD-KNN prediction accuracy across plant species**

Supplementary Figure S1. Effect of neighbour number on HD-KNN prediction accuracy across plant species. Species-specific HD-KNN performance was evaluated across different values of k for Brassicaceae, Poaceae, and the remaining plant species. Red dashed lines show microbial community prediction error measured as Jensen-Shannon divergence (JSD), green dashed lines show prediction error measured as mean absolute error (MAE), and blue solid lines show the average plant nuclear ITS-derived host-relatedness distance, measured as normalized Hamming distance, between each held-out species and its k nearest neighbours. Lower JSD and MAE values indicate better prediction accuracy, whereas lower Hamming distances indicate closer host-relatedness among selected neighbours. The figure illustrates that species with closer neighbours, particularly within densely sampled Brassicaceae and Poaceae groups, generally showed improved HD-KNN performance, supporting the local-neighbour structure of the model.


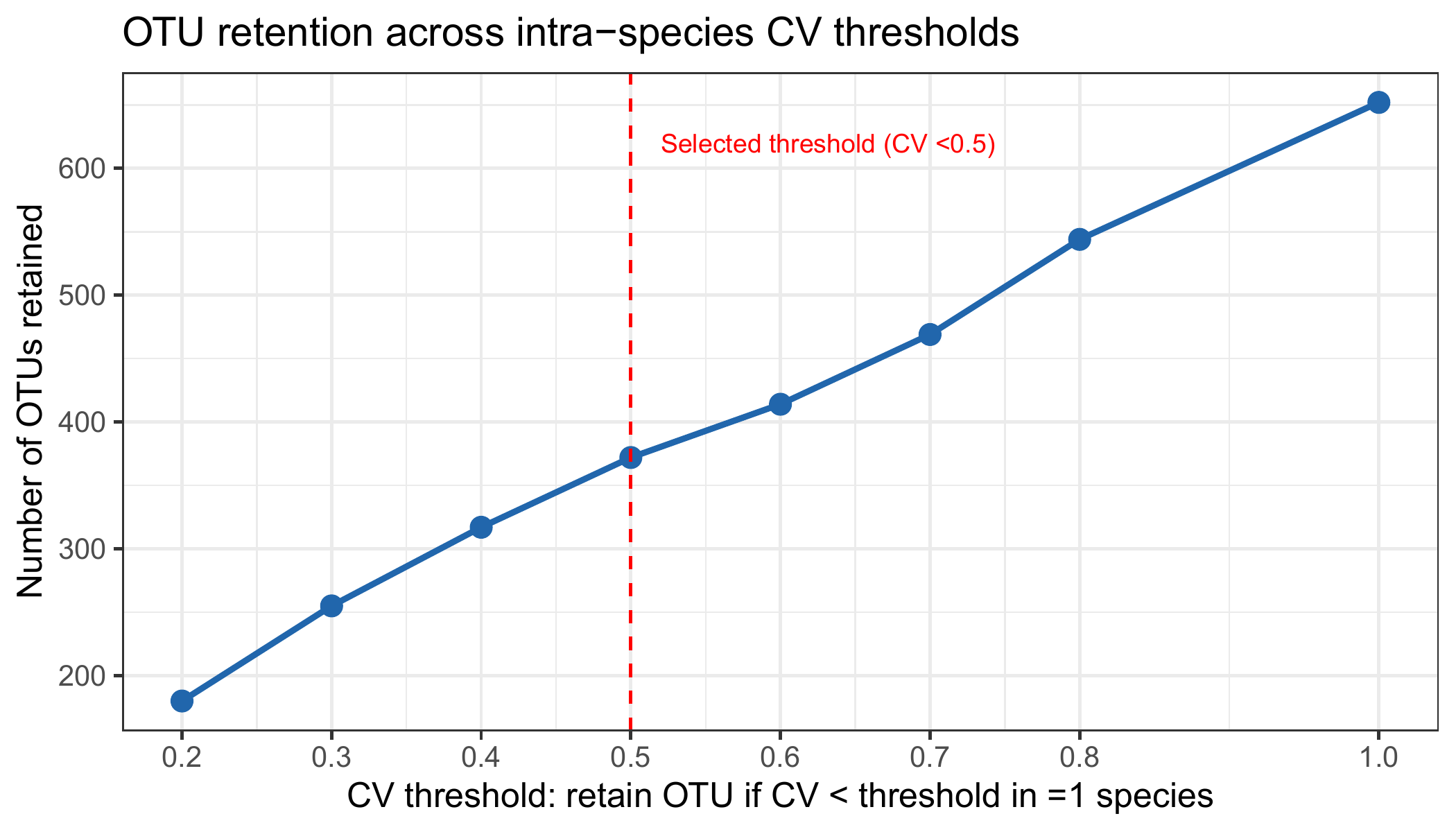
**Supplementary Figure S2a. Number of OTUs retained across intra-species CV thresholds**

*The number of OTUs retained under different intra-species coefficient of variation (CV) thresholds. OTUs were retained if their CV was below the given threshold in at least one plant species. The selected threshold, CV <0.5, retained 372 OTUs from the initial 11,739 OTUs. Relaxing the threshold increased the number of retained OTUs gradually, indicating that the selected threshold does not represent an abrupt cutoff.*


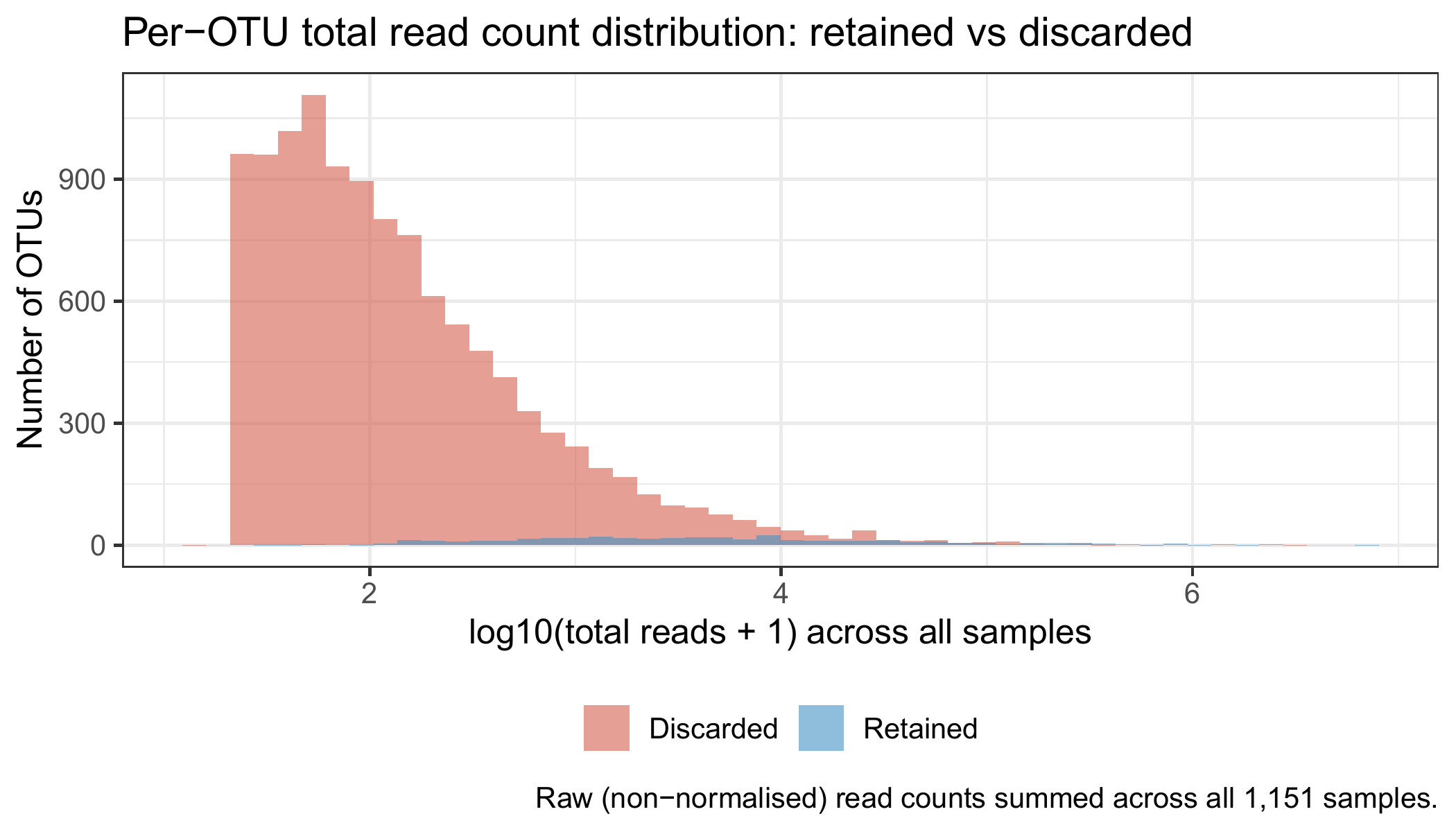
**Supplementary Figure S2b. Read-count distribution of retained and discarded OTUs**

*Distribution of raw read counts for OTUs retained by the CV <0.5 filter compared with discarded OTUs. The retained 372 OTUs represented only 3.2% of total OTU richness but accounted for 64.58% of total raw sequencing reads, indicating that the filtered target space captured a numerically dominant fraction of the seed-associated bacterial community.*


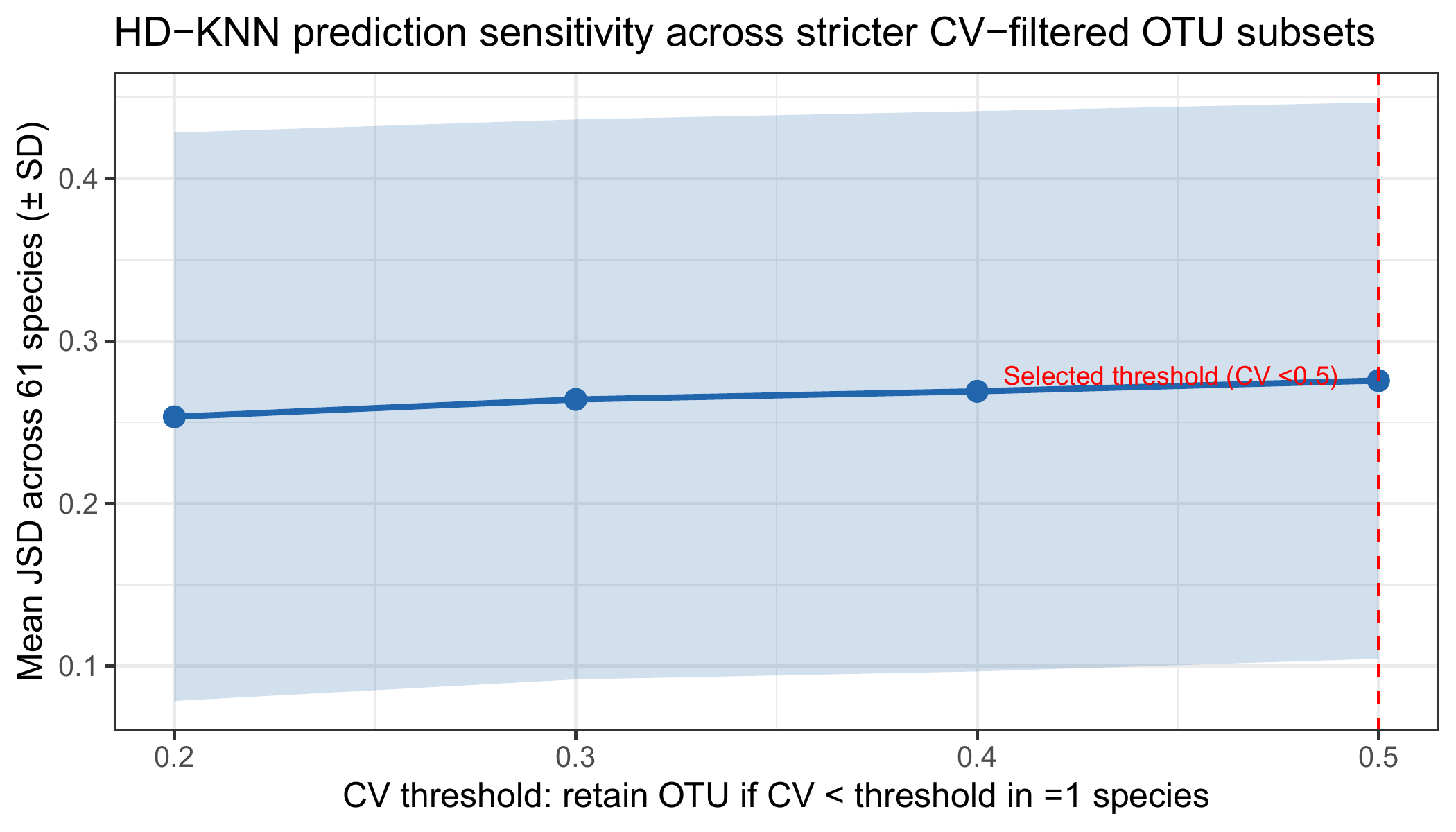
**Supplementary Figure S2c. HD-KNN prediction performance across stricter CV-filtered OTU subsets**

*Mean Jensen-Shannon divergence (JSD) for HD-KNN predictions computed across OTU subsets retained under stricter intra-species CV thresholds. JSD was evaluated using the original benchmark predictions for OTUs contained within the 372-OTU benchmarked target space. Mean JSD changed gradually from 0.2534 ± 0.1749 at CV <0.2 to 0.2757 ± 0.1712 at the selected threshold of CV <0.5. Thresholds above CV <0.5 admit additional OTUs that were not included in the original benchmark and therefore were not interpreted as full model sensitivity analyses.*

**
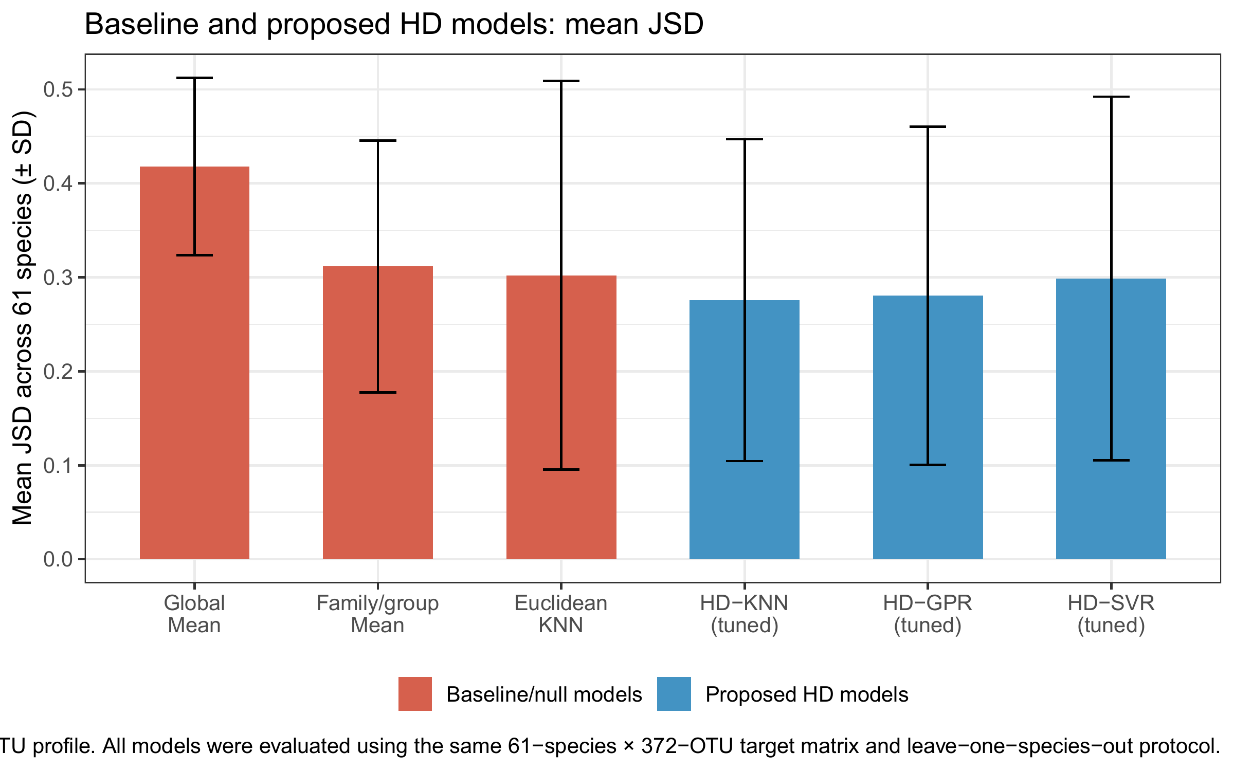
Supplementary Figure S3. Baseline and proposed HD model comparison**

*Mean Jensen-Shannon divergence (JSD) across 61 plant species for baseline/null models and the proposed Hamming-distance-based models. Lower JSD indicates better prediction. Error bars indicate standard deviation across species. All models were evaluated using the same 61-species × 372-OTU target matrix and leave-one-species-out protocol.*


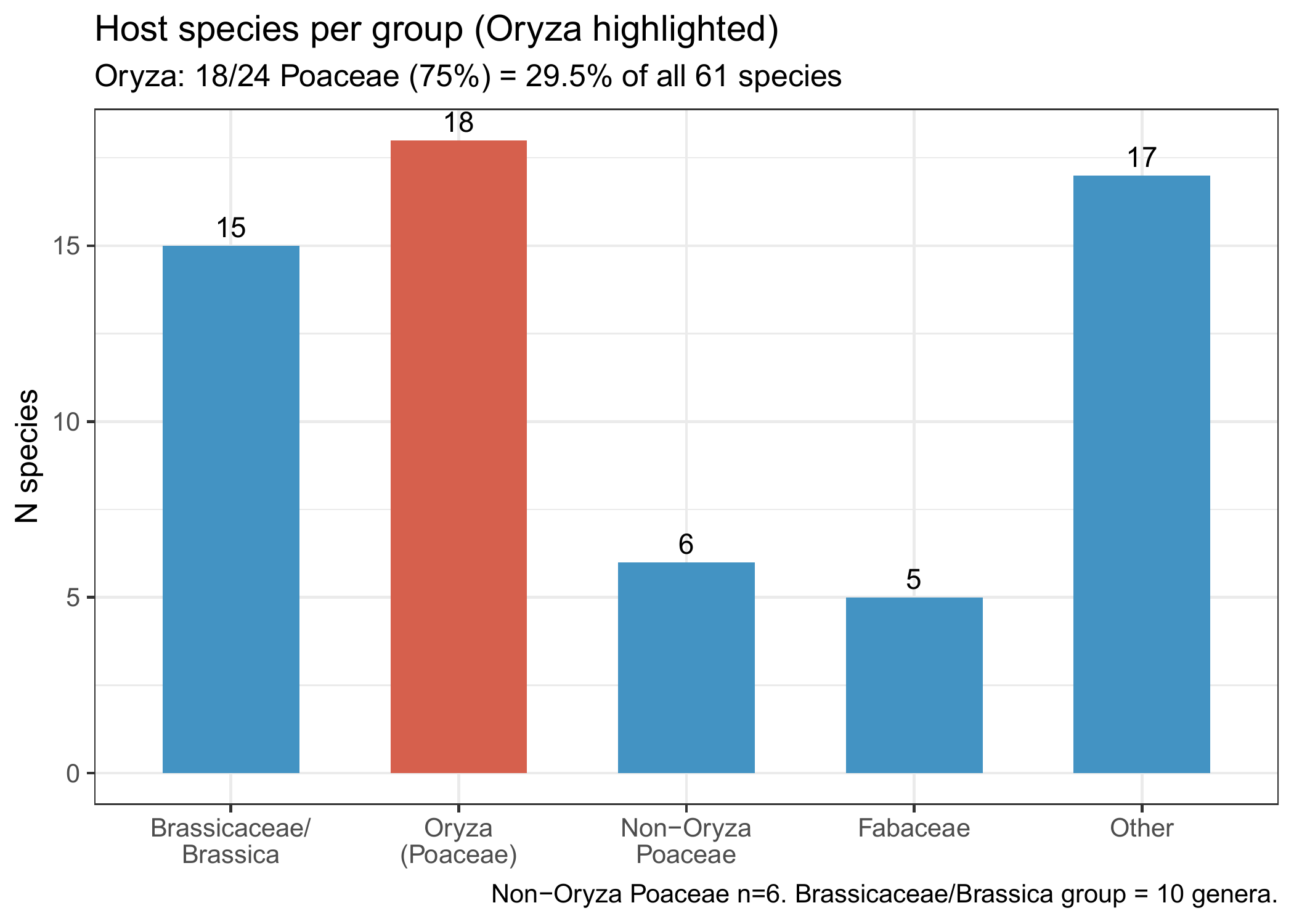
**Supplementary Figure S4. Host species representation across major groups**

*Number of plant species included in each host group used for the Oryza-exclusion sensitivity analysis. The dataset contained 61 plant species in total: 15 Brassicaceae species, 18 Oryza species, 6 non-Oryza Poaceae species, 5 Fabaceae species, and 17 Other species. Oryza represented 18 of 24 Poaceae species, corresponding to 75% of Poaceae and 29.5% of all analysed plant species, highlighting the uneven host representation in the dataset.*


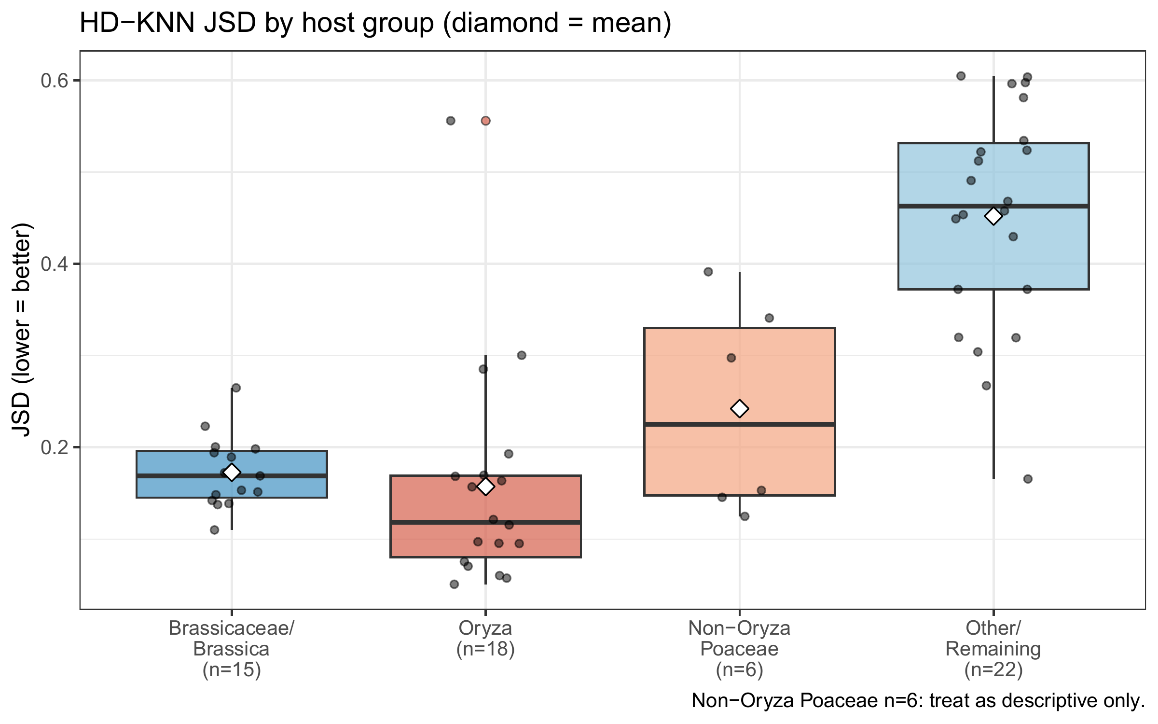
**Supplementary Figure S5. HD-KNN prediction performance across host groups with *Oryza* separated from non-*Oryza* *Poaceae***

*Jensen-Shannon divergence (JSD) values are shown for individual plant species grouped as Brassicaceae, Oryza, non-Oryza Poaceae, and Other/Remaining species. Lower JSD indicates better prediction. Boxplots show the median and interquartile range, points represent individual species, and white diamonds indicate group means. Oryza species showed lower prediction error than non-Oryza Poaceae, while the Other/Remaining group showed the highest error. Because the non-Oryza Poaceae group contained only six species, this comparison should be interpreted descriptively.*
